# Ceramics for Dental Applications: A Review

**DOI:** 10.3390/ma3010351

**Published:** 2010-01-11

**Authors:** Isabelle Denry, Julie A. Holloway

**Affiliations:** The Ohio State University College of Dentistry, 305 W 12^th^ Avenue, Columbus, OH 43210, USA; E-Mail: holloway.3@osu.edu (J.A.H.)

**Keywords:** dentistry, ceramic, crystalline phase, alkali silicates, zirconia

## Abstract

Over the past forty years, the technological evolution of ceramics for dental applications has been remarkable, as new materials and processing techniques are steadily being introduced. The improvement in both strength and toughness has made it possible to expand the range of indications to long-span fixed partial prostheses, implant abutments and implants. The present review provides a state of the art of ceramics for dental applications.

## 1. Introduction

Due to the unsurpassed mechanical properties of partially stabilized zirconia, its introduction to the dental market, almost a decade ago, considerably expanded the range of applications of ceramics in dentistry, a field where they are classically in demand due to their chemical inertness and a wide combination of optical properties, allowing excellent esthetics. Even though the current trend is toward the development of all-ceramic systems, ceramics are still widely used for veneering metallic frameworks for dental restorations. Concurrently, ceramic posts, abutments and implants are now becoming available. Dental ceramics can be classified according to their crystalline phase and fabrication technique (Table 1). This classification is constantly evolving with latest developments leading to the combination of several fabrication techniques and core/veneering ceramic systems, with the ultimate goal of achieving adequate strength and toughness, optimal esthetics and long-term *in vivo* performance. The present review attempts to summarize the various dental ceramic systems from a chemical aspect and give some insight into the relationship between structure and properties of dental ceramics.

**Table 1:** Classification of Dental Ceramics.

	Fabrication technique	Crystalline phase
**Metal-ceramics**	Sintering	Leucite
	Heat-pressing on metal	Leucite, leucite & fluorapatite
**All-ceramics**	Sintering	Leucite
	Heat-pressing	Leucite, lithium disilicate
	Dry pressing and sintering	Alumina
	Slip-casting & glass infiltration	Alumina, spinel, alumina-zirconia (12Ce-TZP)
	Soft machining & glass-infiltration	Alumina, alumina-zirconia (12Ce-TZP)
	Soft machining & sintering	Alumina, zirconia (3Y-TZP)
	Soft machining, sintering & heat-pressing	Zirconia/fluorapatite-leucite glass-ceramic
	Hard machining	Sanidine, leucite
	Hard machining & heat treatment	Lithium disilicate

## 2. Metal-Ceramics

**Figure 1:**
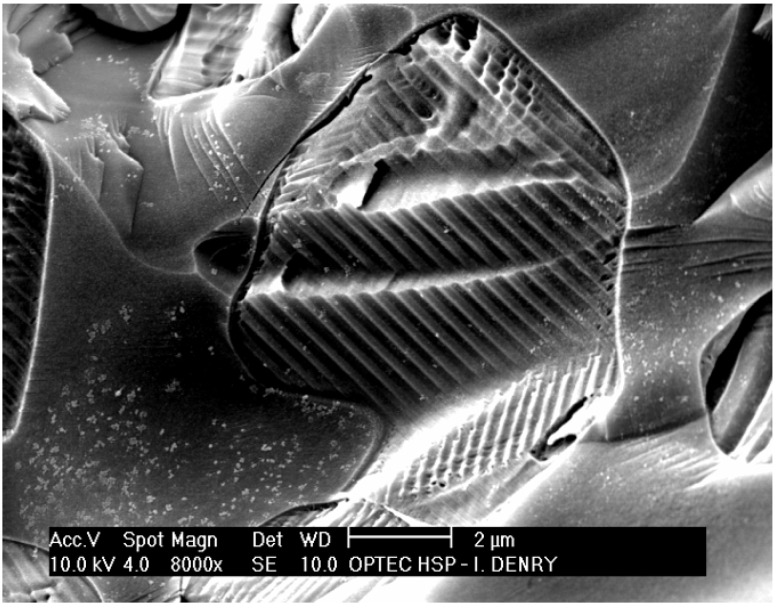
Leucite crystal in a veneering dental ceramic (fractured surface).

Metal-ceramic systems for dental restorations have been available since the 1960s. They rely on the application and firing of a veneering ceramic onto a metal substructure to produce an esthetically acceptable restoration. Veneering ceramics for metal-ceramic restorations -commonly named feldspathic porcelains- are usually leucite-based [1]. Feldspar-derived glass alone exhibits a low coefficient of thermal expansion, around 8.6 × 10^-6^/°K [2]. The addition of leucite to feldspar glass led to the production of veneering ceramics with a coefficient of thermal expansion compatible with that of the metal substructure. Leucite (KAlSi_2_O_6_) is a potassium alumino-silicate that exhibits a tetragonal structure at room temperature and undergoes a displacive phase transformation from tetragonal to cubic at 625 °C, accompanied with a volume expansion of 1.2% [3,4]. This results in a high coefficient of thermal expansion (20 to 25 × 10^-6^/°K) [5]. The transformation has been reported to start at temperatures as low as 400 °C in feldspathic dental porcelains [6,7]. Leucite can be obtained by incongruent melting of naturally-occurring feldspar at temperatures between 1150 and 1530 °C [8]. By varying the proportion of leucite to feldspar glass, the coefficient of thermal expansion can be precisely adjusted by the manufacturer. As shown in Figure 1, leucite crystals often exhibit lamellar twinning due to the phase transformation, so as to minimize macroscopic strain [9].

**Figure 2:**
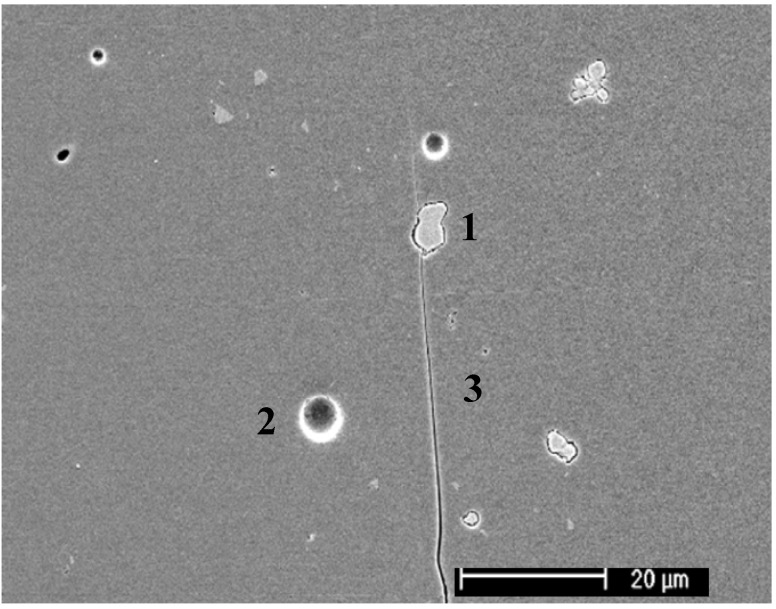
Defects in a low crystallinity veneering ceramic. (1) Inclusion; (2) Pore; (3) Crack.

**Figure 3:**
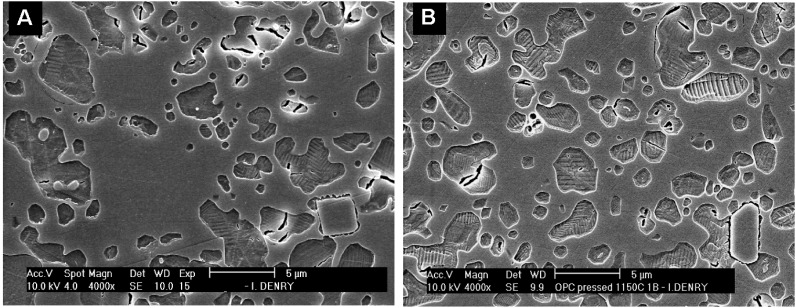
(a) Feldspathic dental porcelain. (b) First generation heat-pressed leucite-reinforced ceramic (3b) Reproduced with permission from [17].

Feldspathic dental porcelains usually contain between 15 and 25 vol % leucite. This amount is adjusted so that the coefficient of thermal contraction of the porcelain is slightly lower than that of the metal, in order to place the ceramic under slight compression [10]. Veneering ceramics for dental restorations are classically sintered under vacuum in order to reduce the porosity of the final product [11,12]. Unfortunately, pores are not the only defects found in veneering ceramics, as shown in Figure 2, cracks and inclusions are also present. The microstructure of a leucite-containing dental porcelain is shown in Figure 3A. Leucite crystals exhibit a polygonal shape with an overall diameter between 1 and 5 micrometers, larger clusters are sometimes observed. Due to the large difference in coefficient of thermal expansion between the leucite crystals and the surrounding glassy matrix, radial tensile stresses and tangential compressive stresses develop within and around the crystals upon cooling, leading to the formation of microcracks [10]. Decoupling of the leucite particles from the matrix has been reported, potentially affecting the coefficient of thermal expansion of the ceramic [13]. Multiple firings and slow cooling rates have also been shown to affect the amount of leucite in dental porcelains, thereby altering their thermal expansion behavior [14,15]. The mechanical properties of feldspathic porcelains are the lowest of ceramic materials used in dentistry and dominated by the large amount of glassy phase [16].

Considering that metal-ceramic dental restorations have been used in dentistry for more than four decades, their overall performance can be considered as quite successful. This is mainly due to sustained efforts by manufacturers to improve the quality of the materials offered, particularly in terms of crystal size and optical properties, such as opalescence. Metal-ceramic technology is challenging, and optimal esthetics can only be achieved by skilled technicians. Nevertheless, it was estimated in 2005 that more than 50% of all dental restorations fabricated were metal-ceramics [18].

## 3. All-Ceramic Systems

Driven by a debatable need for metal-free restorations, the evolution of all-ceramic systems for dental restorations has been remarkable in last three decades. Processing techniques novel to dentistry have been developed, such as heat-pressing, slip-casting, and Computer Aided Design-Computer-Aided Machining (CAD-CAM). Concurrently, all-ceramic materials have been developed to match dental requirements, offering increasingly greater performance from a mechanical standpoint. As opposed to metal-ceramics, all-ceramics contain a significantly greater amount of crystalline phase, from about 35 to about 99 vol %. This higher level of crystallinity is responsible for an improvement in mechanical properties through various mechanisms, such as crystalline reinforcement or stress-induced transformation. Unfortunately, higher crystallinity is also associated with higher opacity, which is not always desirable for dental ceramics. As an example, zirconia ceramics such as 3Y-TZP (3 mol % Yttria-stabilized Tetragonal Zirconia Polycrystals) offer unsurpassed mechanical properties but are also the most opaque of all-ceramic materials currently available [19]. However, crystallinity is only one of many intrinsic factors contributing to materials performance. Other factors such as crystal size and geometry, modulus of elasticity, phase transformation and thermal expansion mismatch between crystal and glassy phase play a crucial role in determining the final mechanical response of the ceramic.

It should also be kept in mind that when it comes to all-ceramic systems, extrinsic factors such as working conditions play a major role in the long-term performance of the material. The oral environment assembles a set of challenging working conditions that include humidity, acidic or basic pH, cyclic loading and peak loads that can reach extremely high levels when hard objects are accidentally encountered during mastication. A humid environment is susceptible to lead to stress corrosion and catastrophic failure in ceramic materials including a glassy phase [20]. The same is true for some highly crystalline materials such as 3Y-TZP, which has been shown to undergo microstructural degradation in a humid environment at relatively low temperatures [21,22,23]. It is therefore generally accepted that tests performed in a humid environment and under cyclic loading are needed to provide valuable information on the long-term performance of dental ceramics [24].

### 3.1. Heat-pressed ceramics

The popularity of heat-pressed ceramics relies on the ability to use the lost-wax technique to produce dental ceramic restorations. Dental technicians are usually familiar with this technique, commonly used to cast dental alloys. In addition, the equipment needed to heat-press dental ceramics is relatively inexpensive. The first generation of heat-pressed dental ceramics contains leucite as reinforcing crystalline phase. The second generation is lithium disilicate-based.

First generation heat-pressed ceramics contain between 35 and 45 vol % leucite as crystalline phase [25]. A representative microstructure is shown in Figure 3B. Flexural strength and fracture toughness values that are about two times higher than those of feldspathic porcelains [26]. This increase in strength and toughness was explained by dispersion of fine leucite crystals from the heat-pressing process [27]. In addition, as pointed out earlier, tangential compressive stresses develop around the crystals upon cooling, due to the difference in thermal expansion coefficients between leucite crystals and glassy matrix. These stresses can contribute to crack deflection and improved mechanical performance [28]. It should be noted, however, that coalescence of microcracks can also cause decoupling of the crystals from the matrix and lead to a degradation in strength and fracture toughness [29]. The presence of about 9% porosity should also be considered, when analyzing the mechanical properties of this system [27]. Further work revealed that the flexural strength of these ceramics was significantly improved after additional firings, due to additional leucite crystallization [30]. Another study examined the phase stability of leucite in this system [31], and revealed that tetragonal leucite is the stable phase at temperatures and durations needed for dental laboratory processing.

**Figure 4:**
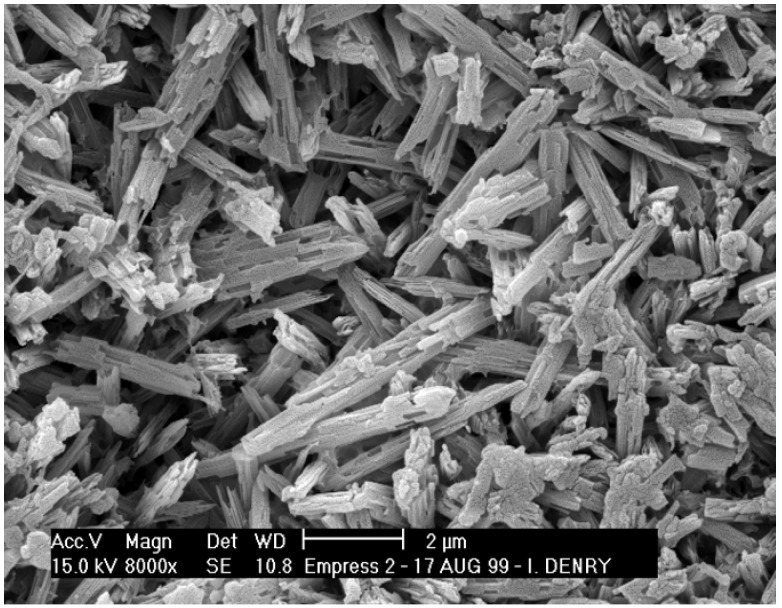
Microstructure heat-pressed lithium disilicate glass-ceramic.

**Figure 5:**
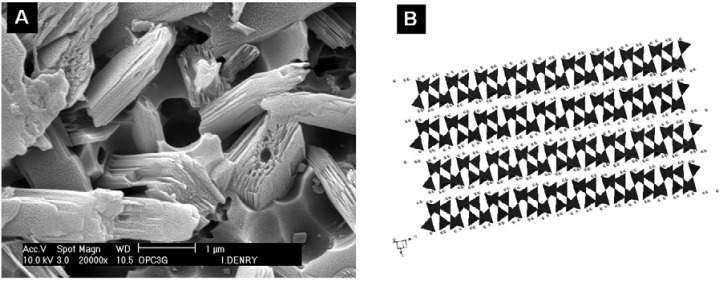
(a) Interlocked crystals in lithium disilicate glass-ceramic; (b) Crystallographic structure of Li_2_Si_2_O_5_, layers are composed of SiO_4_ tetrahedra sharing corners, Li atoms in gray.

Second generation heat-pressed ceramics contain about 65 vol % lithium disilicate as the main crystalline phase, with about 1% porosity [27]. Lithium disilicate glass-ceramics have been extensively studied [32,33,34,35,36,37]. All studies seem to agree that the mechanisms leading to the crystallization of lithium disilicate in these systems are somewhat complex, due to the presence of nanosized crystal phases [32]. High temperature X-ray diffraction studies revealed that both lithium metasilicate (Li_2_SiO_3_) and cristobalite (SiO_2_) form during the crystallization process, prior to the growth of lithium disilicate (Li_2_Si_2_O_5_) crystals [37]. The final microstructure consists of highly interlocked lithium disilicate crystals, 5 μm in length, 0.8 μm in diameter (Figure 4). Borom *et al*. [32], remarked that the thermal expansion mismatch between lithium disilicate crystals and glassy matrix is likely to result in tangential compressive stresses around the crystals, potentially responsible for crack deflection and strength increase. The interlocked microstructure and layered crystals are also likely to contribute to strengthening (Figure 5). Crack propagation is easy along the cleavage planes, but more difficult across the planes, leading to multiple crack deflections due to an array of crystal orientations. Several authors reported crystal alignment after heat-pressing lithium disilicate glass-ceramics [27,38,39]. This result can be expected to some extent due to the high aspect ratio of the crystals, and is likely to affect differently the mechanical properties in directions parallel or perpendicular to crystal alignment, with higher resistance to crack propagation in the direction perpendicular to crystal alignment.

Overall, lithium disilicate glass-ceramics for all-ceramic restorations have performed well. Their strength is more than twice that of first generation leucite-reinforced all-ceramics and their good performance has led to their expanded use to restorations produced by machining.

### 3.2. Dry-pressed and sintered ceramics

Densely sintered alumina-based ceramics produced by dry pressing, followed by sintering have been available since the early 1990s and are still currently used. The technique involves computer-aided production of an enlarged die in order to compensate for sintering shrinkage (12 to 20%). Dry pressing and sintering of a high purity alumina-based core ceramic is then performed at high temperature (1550 °C). This leads to a highly crystalline ceramic with a mean grain size of about 4 micrometers and a measured flexural strength of 601 ± 73 MPa [40,41,42]. All production steps are carefully controlled by the manufacturer. The high-strength core is then veneered with translucent porcelain to achieve adequate esthetics. Clinical results have demonstrated an excellent *in vivo* performance at 15 years [43]. The same technology is also available for zirconia-based core ceramics.

### 3.3. Slip-cast ceramics

Slip-cast ceramics for dental restorations were introduced in the 1990s. A porous infrastructure is produced by slip-casting, sintered, and later infiltrated with a lanthanum-based glass, producing two interpenetrating continuous networks, one composed of the glassy phase and the other being the crystalline infrastructure. Three crystalline phases are available, namely alumina (Al_2_O_3_), spinel (MgAl_2_O_4_) and zirconia-alumina (12 Ce-TZP-Al_2_O_3_).

**Figure 6:**
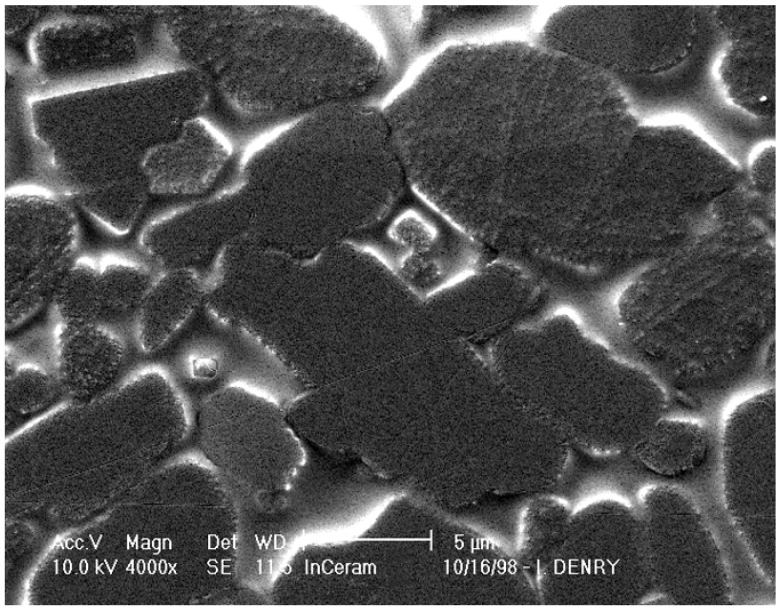
Microstructure of alumina slip-cast ceramic.

Alumina-based slip-cast ceramics contain 68 vol % alumina, 27 vol % glass and 5 vol % porosity [27]. The microstructure consists of blocky alumina grains of various sizes and shapes (Figure 6). Evidence of grain pull-out, bridging and crack deflection was reported with this type of ceramic [27], indicative of efficient crystalline reinforcement, and accounting for mechanical properties in the range of heat-pressed lithium disilicate glass-ceramics. It has also been suggested that the coefficient of thermal expansion mismatch between the alumina crystals and the infiltration glass could contribute to strengthening due thermal residual stresses. The presence of large alumina crystals with a high refractive index, and a non-negligible amount of porosity, account for some degree of opacity in this all-ceramic system. Spinel-based slip-cast ceramics offer better translucency [44], similar to that of lithium disilicate heat-pressed ceramics, at the expense of mechanical properties [45,46].

**Figure 7:**
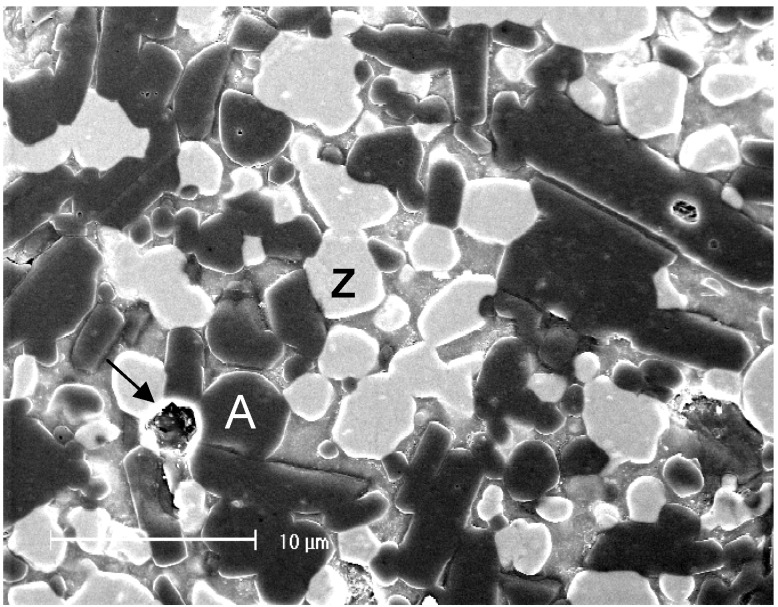
Microstructure of zirconia-toughened alumina slip-cast ceramic. A: alumina grains, Z: zirconia grains, Arrow indicates pore.

Zirconia-toughened alumina slip-cast ceramics comprise 34 vol % alumina and 33 vol % of 12 mol % ceria-stabilized zirconia (12Ce-TZP). The glass phase represents approximately 23 vol % of the final product, with about 8 vol % residual porosity [47,48]. The microstructure is shown in Figure 7, with large alumina grains in darker contrast, smaller 12Ce-TZP grains in brighter contrast, and some porosity visible as well. The dual crystalline reinforcement in this system allows two types of strengthening mechanisms:

(1) The stress-induced transformation in zirconia grains produces compressive stresses within the transformed grains and surrounding glassy matrix, as well as circumferential tensile stresses around the grains, accompanied by microcrack nucleation. Keeping in mind that transgranular fracture is difficult in zirconia, this represents an efficient strengthening mechanism.

(2) Crack deflection, contact shielding and crack bridging are expected from the presence of large alumina grains [47].

The combination of these two strengthening mechanisms explains why alumina-zirconia slip-cast ceramics offer the highest flexural strength and fracture toughness of all slip-cast ceramics [49].

### 3.4. Machined ceramics

Computer Aided Design/Computer Aided Design (CAD/CAM) technology was introduced in dentistry by Duret in the early 70’s [50]. The technology was originally intended for fully sintered ceramic blocs (hard machining), it has now been expanded to partially sintered ceramics (soft machining), that are later fully heat treated to ensure adequate sintering.

#### Hard machining

Early materials included a machinable fluormica glass-ceramic exhibiting a classic “house-of-cards” microstructure of interlocking mica platelets. Cleavage planes offered by the mica crystals gave the ceramic an excellent machinability [51]. Currently, fully sintered ceramic materials available for CAD/CAM hard machining of dental restorations include feldspar-based, leucite-based and lithium disilicate-based ceramics.

**Figure 8:**
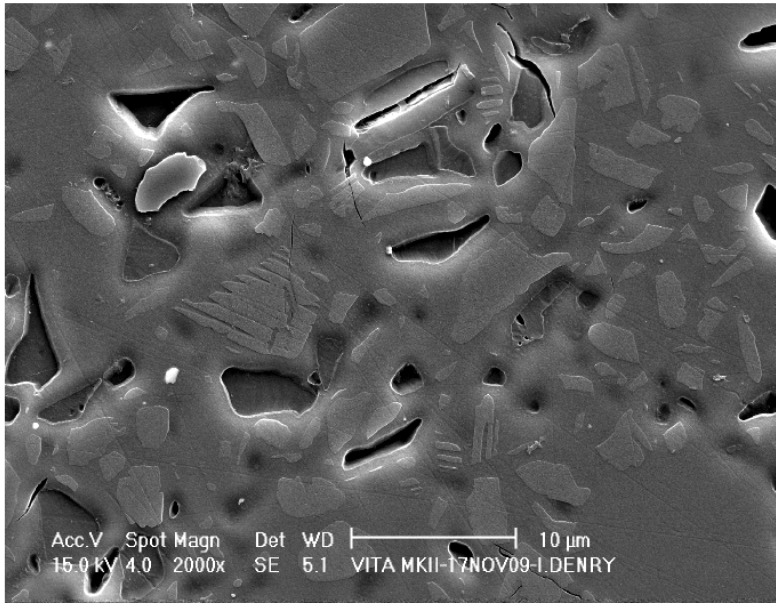
Microstructure of a fully sintered machinable feldspar-based ceramic.

The microstructure of the feldspar-based material is shown in Figure 8. Polygonal sanidine [(Na,K) AlSi_3_O_8_] crystals (2–10 μm in diameter) appear in lighter contrast within the glassy matrix. A few microcracks are present, possibly indicating some degree of thermal expansion mismatch between some of the crystals and the glassy matrix. The amount of crystalline phase in this material is about 30 vol % [52,53].

A leucite-reinforced ceramic is also available as machinable blocs for CAD/CAM restorations. This material is similar in microstructure and mechanical properties to the first generation leucite-reinforced pressable ceramics. Machining of fully sintered ceramics typically creates significant tool wear and residual surface flaws that could, in the long-term, be detrimental to the *in vivo* performance of the ceramic [54].

**Figure 9:**
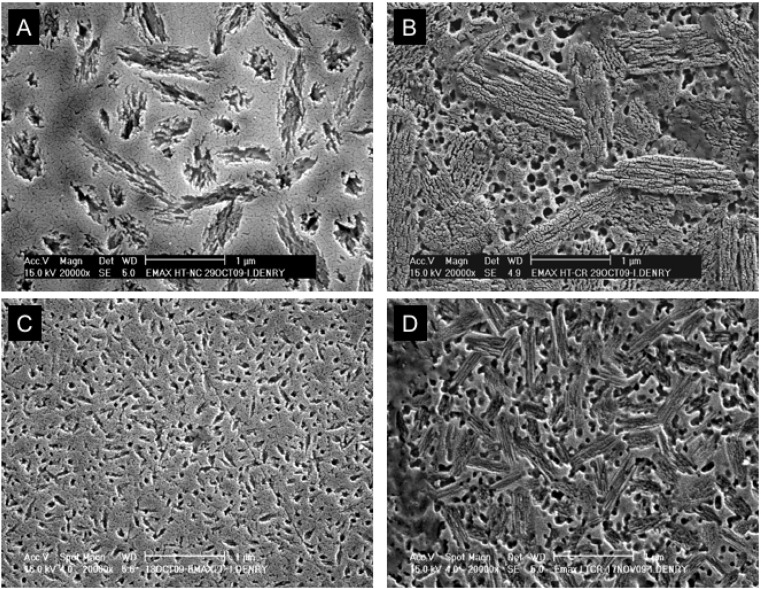
(a) Partially crystallized High Translucency (HT) lithium silicate machinable ceramic. (b) Fully crystallized HT lithium disilicate ceramic. (c) Partially crystallized Low Translucency (LT) lithium silicate machinable ceramic. (d) Fully crystallized LT lithium disilicate ceramic.

An elegant approach to CAD/CAM machining of fully sintered ceramics was proposed with the introduction of partially crystallized ceramics in the lithium silicate system. The ceramic blocs are partially crystallized and contain both lithium metasilicate (Li_2_SiO_3_) crystals and lithium disilicate (Li_2_Si_2_O_5_) crystal nuclei. In this state, the ceramic is easy to machine and exhibits moderate strength (130 MPa, according to manufacturer’s data). Depending on the crystallization pre-treatment of the ceramic blocs, two levels of translucency can be obtained. The high translucency (HT) material contains fewer and larger crystals of lithium metasilicate in the pre-crystallized state (Figure 9A), while the low translucency (LT) material contains a higher density of smaller crystals (Figure 9C).

After full crystallization heat treatment at 850 °C for 10 minutes, the HT ceramic exhibits layered lithium disilicate crystals (1.5 × 0.8 μm) in a glassy matrix (Figure 9B). Highly soluble lithium phosphate spherical crystals appear as spherical pores. The fully crystallized LT ceramic exhibits a high density of small (0.8 × 0.2 μm) interlocked lithium disilicate crystals, together with spherical pores, also interpreted as lithium phosphate crystals (Figure 9D). X-ray diffraction data (not presented) was used to confirm these findings. The flexural strength after full crystallization heat treatment is 360 MPa, according to manufacturer’s data. One study reported a flexural strength (3-point bending) of 134 ± 27 MPa in the as-received state and 262 ± 88 MPa after full crystallization [55].

#### Soft machining

**Figure 10:**
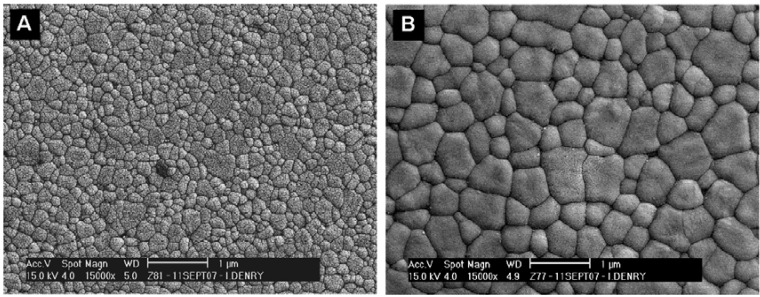
3Y-TZP ceramic sintered at (a) 1300 °C for 2 hours and (b) 1500 °C for 2 hours.

Soft-machining of partially sintered zirconia ceramic blocs by CAD/CAM technology, to produce dental restorations was proposed in 2001 after intensive research work [56,57]. The design compensates for the volume shrinkage that will later occur during sintering of the zirconia blocs (about 25%). The partially sintered blocs are easy to mill, which leads to substantial savings in time and tool wear. The type of zirconia used in this technology is biomedical grade tetragonal zirconia stabilized with 3 mol % yttria (3Y-TZP) [58]. Unalloyed zirconia is monoclinic at room temperature and tetragonal above 1170 °C [59]. The tetragonal to monoclinic transformation (t→m) is associated with a substantial volume increase (~4.5%). The high temperature tetragonal form can be stabilized at room temperature by addition of various oxides, including yttria, ceria, calcia or magnesia [59,60,61]. Partially stabilized tetragonal zirconia exhibits phase transformation toughening, which involves the transformation from tetragonal to monoclinic phase at the crack tip, associated with a volume increase, thereby creating compressive stresses. This mechanism is efficient in preventing further crack propagation and is responsible for the outstanding mechanical properties of partially stabilized zirconia [61,62,63]. Of interest is the fact that the stability and therefore the mechanical properties of 3Y-TZP strongly depend on its grain size [64,65]. Above a critical grain size, 3Y-TZP is less stable and more susceptible to spontaneous transformation while smaller grain sizes are associated with a lower transformation rate [66,67]. Grain size is determined by the sintering conditions and particularly the sintering temperature and duration. As shown in Figure 10, higher temperatures and longer durations lead to larger grain sizes. Currently available 3Y-TZP ceramics for soft machining of dental restorations require sintering temperatures varying from 1350 to 1550 °C and durations from 2 to 6 hours, depending on the manufacturer.

These differences could account for slight differences observed in the mechanical properties of the final product. Nevertheless, 3Y-TZP ceramics for dental restorations offer to date the best mechanical properties of all-ceramic core materials currently available. It should be noted, however, that problems such as crazing or cracking at the interface between veneering porcelain and core material have been reported clinically [68,69,70,71,72]. Mechanisms involving destabilization of the tetragonal phase at the interface with the veneering porcelain have been proposed [73].

Since its introduction to dentistry, almost a decade ago, the soft machining technique has been extremely successful. The extensive number of dental publications on zirconia, combined with the large amount of literature published on the various types of zirconia prior to its introduction in dentistry, now provides a large database of information. Several review articles provide state of the art information on zirconia ceramics for biomedical applications [58,60,74,75,76]. Studies evaluating the three-year and five-year performance of 3Y-TZP fixed partial prostheses *in vivo* have recently been published [77,78,79]. These studies point out an excellent success rate but a lower survival rate due to complications such as secondary caries and chipping of the veneering ceramic [77]. However, the overall excellent performance of 3Y-TZP restorations processed by the soft machining technique, followed by sintering, has led to its extension to alumina-based ceramics and its combination with other processing techniques such glass-infiltration and heat-pressing.

### 3.5. Manufactured zirconia dental abutments and implants

With the success of zirconia as a dental restorative material, manufactured 3Y-TZP abutments and implants have recently been introduced on the dental market. A recent review on ceramic dental implants concluded that the currently available clinical data is “not sufficient to recommend ceramic implants for routine clinical use” [80]. Concerns with using 3Y-TZP dental abutments and implants rely on the fact hat the material is in contact with biological fluids. As mentioned previously, it is well established that zirconia is susceptible to low temperature degradation [21,23]. A substantial amount of scientific literature is available on the topic, since zirconia has been used for at least two decades to manufacture femoral heads [22,81,82,83,84]. Low temperature degradation of 3Y-TZP involves microstructural changes such as grain pull-out, microcracking and surface roughening [82,85,86]. The ISO standard for Y-TZP implants recommends that the amount of monoclinic phase after accelerated aging for 5 hours be less or equal to 25%, as determined by X-ray diffraction [87]. It has also been demonstrated that surface finish and residual surface stresses strongly influence the response of 3Y-TZP to low temperature degradation [88]. It was pointed out that a significant amount of surface roughening and damage can occur in Y-TZP, even in materials containing less than 25% monoclinic phase after aging for 5 hours. Careful consideration should be given to the use of zirconia for dental abutments, particularly since dental abutments undergo some degree of loading through tightening of a metal abutment screw. Perhaps, at the very least, the properties of Y-TZP dental implants should be considered in light of the established ISO standard for Y-TZP biomedical implants [87]. Meanwhile, a considerable amount of research is being conducted with the aim of developing ceramics for dental and biomedical applications with improved reliability [89,90,91]. This effort has led to the successful production of zirconia/alumina ceramic composites, consisting of either zirconia-toughened alumina (ZTA) or alumina-toughened zirconia (ATZ), depending on the proportion of the main component. These advanced composites exploit the transformation toughening capabilities of zirconia while being less susceptible to low temperature degradation in biological fluids. Ceria-stabilized zirconia/alumina nanocomposites for dental applications have been shown to exhibit high flexural strength (1422 ± 60 MPa), high reliability and an excellent resistance to low temperature degradation [92,93]. Further research is needed to evaluate the long-tem *in vivo* performance of these composites in the oral environment.

## 4. Conclusions

The technological evolution of dental ceramics has been remarkable over the past four decades. From feldspathic porcelains to zirconia-based all-ceramics, tremendous progress has been made in terms of mechanical performance, with a ten-fold increase in flexural strength and fracture toughness. Common important characteristics of all-ceramic systems, such as the proportion of glassy phase and amount of porosity, both influence optical and mechanical properties. Residual stress states between crystalline phases and glassy matrix, as well as microcracking also play a key role in the development high strength ceramics. The two most recently introduced all-ceramic systems (hard machined lithium disilicate and soft machined 3Y-TZP) are excellent examples of successful material development to match specific requirements of dental restorations. Table 2 provides a summary and some examples of ceramic-based systems available for dental restorations.

**Table 2:** Systems available for metal-ceramic and all-ceramic dental restorations.

Processing Method	Crystalline phase	Crystallinity (%)	Strength (MPa)	Brand	Manufacturer
Sintered on metal substructure	Leucite	15–25	61 ± 5 [16]	Ceramco^®^3	Dentsply
Heat-pressed	Leucite	≈ 35	106 ± 17 [27]	IPS Empress^®^	Ivoclar
Heat-pressed	Lithium disilicate	65	306 ± 29 [27]	IPS Empress^®^ Eris	Ivoclar
Dry-pressed and sintered	Alumina	Highly crystalline	607 ± 73 [40]	Procera^®^	Nobel Biocare
Slip-cast & glass-infiltrated or soft machined and glass-infiltrated	Alumina	67–68	594 ± 52 [27]	In-Ceram^®^ Alumina	Vident
Slip-cast & glass-infiltrated or soft machined and glass-infiltrated	Spinel	65–68	378 ± 65 [26]	In-Ceram^®^ Spinell	Vident
Slip-cast & glass-infiltrated or soft machined and glass-infiltrated	12 Ce-TZP-alumina	67	630 ± 58 [47]	In-Ceram^®^ Zirconia	Vident
Soft-machined & sintered	3Y-TZP	Highly crystalline	1087 ± 173 [94]	Cercon^®^	Dentsply
Soft-machined & sintered	Alumina	Highly crystalline	700*	In-Ceram^®^ AL	Vident
Hard-machined	Sanidine	≈ 30	122 ± 13 [26]	Vitablocs^®^ Mark II	Vident
Hard-machined	Leucite	≈ 35	106 ± 17 [27]	IPS Empress^®^ CAD	Ivoclar
Hard-machined & crystallized	Lithium disilicate	65	262 ± 88 [55]	IPS e.max CAD	Ivoclar

* From manufacturer’s data: http://vident.com/products/cadcam/, accessed 12/31/2009
